# The Long Way From Government Open Data to Mobile Health Apps: Overcoming Institutional Barriers in the US Federal Government

**DOI:** 10.2196/mhealth.3694

**Published:** 2014-12-23

**Authors:** Ines Mergel

**Affiliations:** ^1^Maxwell School of Citizenship and Public AffairsDepartment of Public Administration and International AffairsSyracuse UniversitySyracuse, NYUnited States

**Keywords:** mHealth, mobile apps, open data, prizes and challenges

## Abstract

**Background:**

Government agencies in the United States are creating mobile health (mHealth) apps as part of recent policy changes initiated by the White House’s Digital Government Strategy.

**Objective:**

The objective of the study was to understand the institutional and managerial barriers for the implementation of mHealth, as well as the resulting adoption pathways of mHealth.

**Methods:**

This article is based on insights derived from qualitative interview data with 35 public managers in charge of promoting the reuse of open data through Challenge.gov, the platform created to run prizes, challenges, and the vetting and implementation of the winning and vendor-created apps.

**Results:**

The process of designing apps follows three different pathways: (1) entrepreneurs start to see opportunities for mobile apps, and develop either in-house or contract out to already vetted Web design vendors; (2) a top-down policy mandates agencies to adopt at least two customer-facing mobile apps; and (3) the federal government uses a policy instrument called “Prizes and Challenges”, encouraging civic hackers to design health-related mobile apps using open government data from HealthData.gov, in combination with citizen needs. All pathways of the development process incur a set of major obstacles that have to be actively managed before agencies can promote mobile apps on their websites and app stores.

**Conclusions:**

Beyond the cultural paradigm shift to design interactive apps and to open health-related data to the public, the managerial challenges include accessibility, interoperability, security, privacy, and legal concerns using interactive apps tracking citizen.

## Introduction

### The Federal Government and Open Data

The Open Government Directive and Digital Government Strategy of the Obama Administration call for innovative approaches to increase participation, collaboration, and transparency of government operations, especially with mobile phone apps [1,2]. At the center of the initial efforts is a website [3], a new platform to publish government datasets, for an overview of the platform see [4]. Federal departments, among them the National Institutes of Health (NIH), the Centers for Disease Control and Prevention (CDC), the Food and Drug Administration, and the Substance Abuse and Mental Health Services Administration under the umbrella of the Department of Health and Human Services (DHHS), move so-called high-value datasets in machine-readable format on to the Internet for public consumption.

The effort to promote the availability and reuse of the datasets is combined with the open innovation platform Challenge.gov, see for example [5,6]. Using a policy instrument called Prizes and Challenges, developer contests such as the Health 2.0 program invite civic hackers as well as professional problem solvers to reuse health-related public sector data and crowdsource solutions in the form of mobile phone apps [2]. Both initiatives are designed to create public awareness, but also to promote external innovations based on citizen needs. While citizen-driven ideas can be highly individual, and the development of mobile apps themselves is usually limited to professionals with highly specialized skills and coding knowledge, in the aggregate they can add value to larger parts of the population and ensure more effective reuse of open data [7]. The invention and acquisition process itself is usually not part of the scope and responsibility of government’s core mission to deliver public health. Government organizations are missing the design skills, need to jump through legal hoops, and in part have to rethink their approach of informing and educating the public given these new types of technological platforms.

The uptake of the release of health-related datasets, the use of contests and prizes to promote the datasets, and ultimately the implementation and promotion of the innovative outcomes such as mobile phone apps is facing significant institutional barriers unique to the public sector context [7]. The top-down political mandate has freed up agency resources to dive into mobile app development, create best practices for commercial as well as private mobile app review processes, and might set new standards for government-wide development of mobile apps, as demanded by the Chief Information Officer (CIO) Council [8]. However, existing rules and regulations need to be taken into account, which challenge the development and release process, especially of health mobile apps.

### Current Use of Mobile Health Apps in the United States Federal Government

The result of these managerial and political developments is a set of currently 33 mobile apps promoted on the Department of Health and Human Services’ mobile website, an overview of all apps is available (see Appendix for DHHS) [9]. The apps were developed and promoted by 12 different agencies that are part of DHHS. Among them are, for example, the Agency for Health Care Research and Quality, the CDC, National Cancer Institute, NIH, National Library of Medicine, and the Centers for Medicare and Medicaid Services.

All apps are either replicating information that is already available on the agency’s website or provide access to a searchable database of symptoms, diseases, or health-related alerts. The content supports the mission of government organizations, most agencies have to inform and educate the public by providing neutral, reliable, and trustworthy information. Similar to previous phases of e-Government development, mobile apps in public health agencies are still at an early maturity level and focus mostly on representing already existing agency content, delivered through an innovative platform [10,11].

There are nine apps that go beyond a mere information and education function, and focus on supporting behavioral change, such as the National Institute of Cancer QuitPal app or the WordWeather app from the National Cancer Institute that help smokers quit their habits. The apps provide health-related information, but also interactive elements, such as calendar functions with reminders, financial goals, or behavior tracking functionalities. There are two of those apps that are targeting a specific audience, and are using gaming technologies. These apps focus on younger patients, such as teenagers, and are designed to offer “a better option for idle hands” [12,13]. As an example, the NIH’s Brrd Brawl App banks on the popularity of the mobile phone app Angry Birds, and tries to attract younger demographics to help defend against what the app calls “cold turkeys” to protect the farms against invading penguins to stay in a “never-ending survival mode” [14].

A set of apps focuses on another specialized audience, health care and emergency management professionals. There are four of the apps that provide health-related alerts about outbreaks or medication warnings that are pushed to health care professionals to keep them up to date. As an example, the CDC’s Influenza app pushes information about the national flu activity out, and provides information about the latest recommendations to help professionals with diagnosis and treatment options. Similarly, the National Library of Medicine provides the LactMed app for nursing mothers to help them understand medical information about medication and dietary supplements. This adds to a series of commercial mobile apps designed for use by health care professionals in their work with patients [15].

Only three apps allow patients to actively share personal health information regarding the phases of their disease with their online social networks, such as sharing to social media sites or sharing of information by email. The goal of the social networking sharing functions is to increase social awareness for the evolution of the patients’ symptoms, treatments, and outcomes in order to increase cognizance, and by extension, social support.

An app focuses on higher levels of e-government, and provides information about direct transactions with government agencies. The Open Payments Mobile for Industry app of the Centers for Medicare and Medicaid Services is designed to help manufacturers keep track of, store, and view financial payments of industry partners. However, the app does not allow for actual mobile payment transactions.

The analysis of the existing government-owned and -promoted mobile health apps shows a surprising trend toward simple provision of government-vetted information. Apps with higher levels of interactions are rare. There are very few opportunities for citizens to directly interact with the content, to track individual health-related behavior, or for bidirectional exchanges with other patients, supporters, or health care professionals. The guiding research question is therefore, what are the drivers, and most importantly, the barriers for the development and adoption of health-related mobile apps in government, and what are the adoption pathways federal agencies follow?

## Methods

### Using an Interpretative Approach

Much of the nature of the research question of intraorganizational institutional factors leading to the development and ultimately the implementation of mobile apps in government is qualitative in nature. It requires narratives to explain the internal decision making processes, the problems public managers encounter when they start to review interactions of patients—and regular citizens—through their personal data in combination with government data. It is therefore necessary to use an interpretative approach to gain a deeper understanding of each individual agency’s context in which it is operating, as well as their specific situations and specialized internal data. The cumulative insights from a variety of agencies facing the same problems as agencies working with health-related personal data help to open the black box of internal managerial decision making.

### Data Collection

The research design relies on an instrumental case study approach to help inform the adoption of mobile apps by government agencies in general [16], and to derive implications for mobile health apps specifically. A qualitative research design helps to understand the meaning of real-world conditions and perspectives of public managers who are tasked with the development and implementation of mobile health apps. While the outcomes are observable on their agencies’ websites, it is impossible for the researcher to adequately understand the internal situational context and emerging legal conditions that have led to the observable Internet practices.

The aim is to explain events leading to outcomes, and let concepts emerge from the insights provided by the interview partners. The sample includes 35 public managers in agencies of the US Federal Government actively involved in the development of mobile apps in 2012-2013. At the time, ten agencies were actively implementing either in-house designed apps, or outsourced the development to their contractors. In addition, 25 federal agencies actively solicited ideas from the public for the reuse of their datasets available on a data sharing platform [17]. While not all agencies expected mobile apps as a result, many civic hackers and professional coders designed mobile apps. The collection of field-based data aims to accurately capture the contextual decisions across the federal government as a whole, and the individual barriers specific agencies were experiencing.

### Data Analysis

The initial open-ended questions were hand-coded using the qualitative data analysis software QSR NVivo [18]. The initial categories that were represented in the interview questions included, historical evolution of the decision to develop mobile health apps, drivers for these decisions and initial barriers for the development, and the subsequent implication process. The transcripts were coded line-by-line, and the social and managerial processes emerged from this coding process. They were then matched to the phases of e-Government adoption [19], and new categories evolved to help explain the current stage of mobile app development in the US Federal Government.

This iterative data analysis process provides the opportunity to integrate different sources of data, interpret the results, and explain the complexity of the field setting and diversity of the participating government agencies. Following Glaser and Strauss [20], the interpretative data analysis process results in explanations of real-world social behavior. Drivers and barriers are derived “bottom-up” in the form of grounded categories and concepts.

## Results

### Three Pathways to Mobile Apps Development

The qualitative data show that public managers follow three different pathways to develop mobile health apps. These pathways include: (1) in-house development and contracting out to external vendors, (2) top-down policy mandates to develop at least two mobile apps, and (3) running government contests to ask the public to solve public management problems and relying on civic hackathons. However, government agencies face significant barriers as soon as mobile apps are developed that have to be addressed before apps are officially confirmed and promoted on the agency’s website. These barriers include: (1) legal terms of use issues with Apple’s mobile store, (2) accessibility and compliance issues, (3) data privacy issues, and (4) security issues.

### Adoption Pathways

#### Pathway 1, In-House Experimentation and Contracting Out

Mobile health app development started at different times and for different needs in the US Federal Government. Some agencies noticed that with the diffusion of smartphones with Internet access, their agencies’ websites had become unusable to view on certain mobile devices, such as iPads. As one of the interview partners explains,

It started because we created a website for teachers to teach with the records of the [agency] in their classrooms. We had lots of requests from teachers who were using the website that they were getting all sorts of iPads in their schools and they were interested in using the website as an app with students.

Interview Partner

The driver was therefore mainly an adjustment to changing technology standards, rather than internal agency needs or an external policy directive.

The goals of many agencies are not specifically to develop an interactive mobile app that provides a new service, instead the native mobile app development oftentimes coincides with the upgrade of the existing agency website design to a responsive mode, for example, using the programming technology markup language HTML5. As an example, one public manager explained that mobile apps are not standalone solutions for his agency. Instead,

We are moving toward HTML5 on all of our major websites, using active code, that can scale nicely to the mobile devices. It is more than just applications for us.

Public Manager

Other agencies already have their own developers in-house who are developing website features to be accessible across several platforms, such as PC, mobile, and tablets. Agencies used their in-house Drupal developers, who are reusing free and open-source code already developed elsewhere, and adjusted it to the needs of the agency. Using in-house resources allows agencies to be flexible and quicker. However, one public manager points out, “I think down the road we might be contracting out the operation in maintenance and future development”.

In other agencies, similar internal initiatives were started by entrepreneurs [21], or internal trailblazers, even ahead of the official Digital Government Directive that directed agencies to invest in mobile accessible websites and mobile apps. As one public manager in one of the public health agencies explains,

One day I was in a doctor’s office, and when you are in the waiting room of a doctor’s office, they give you a form to fill out, they ask you to list all of the medications and the dietary supplements that you take. While I was filling out that form it occurred to me how hard it is to remember everything that you take and to have really accurate information to be able to share with your doctor. So, I came back to the office and I talked to my supervisor and colleagues about this idea of a mobile app that would allow people to track all of the dietary supplements that they take, so that when they went to the doctor’s office, they would have a more accurate list of everything they take, and that would in turn allow for more accurate communication and more complete communication between the patient and the healthcare provider. In addition to that, we also develop fact sheets on dietary supplements that are science-based, and we wanted to increase our outreach. We also decided to put these fact sheets in the app as well.

Public Manager

Here, the app allows for the reuse of already existing scientific evidence that the agency has to distribute to the public, but added an innovative interactive feature that supports patients’ needs beyond the mere display of information that is already available on the agency’s website.

However, these internal, bottom-up initiatives come at a cost, and are oftentimes met with resistance,

I have been trying to push folks to do mobile since the iPhone came out and the department has a lot of the challenges that is within government’s culture. Remember folks have pretty much just been doing websites and putting everything on the web. Now that we are seeing a paradigm shift, it’s kind of hard to folks to say how do we put our stuff on mobile, or how do we start accepting the new paradigm shift. And some folks just might not know what to do. This is new to them.

Public Manager

This paradigm shift involves a cultural change, and oftentimes a change to the approach of how government passively pushes information to a mobile app that goes beyond the information and representation function many agencies see as their main mission. Citizens have long made the shift. A recent Pew study shows that as of 2014, 90% of US American adults own a cell phone, 58% own a smartphone, and 42% own a tablet [22].

Other agencies adjusted more easily to the paradigm shift,

[...] because we had very strong support from the highest level of the organization, that made it very helpful. So for the first year to get this done, we didn’t get a lot of pushback other than from other business areas, because we are including phone numbers in the tool. And, politically a lot a people did not know about it because it was done at a very high level and very quietly, because we wanted to get it out the door quickly. We had the support of the CCO, one of the deputy commissioners, and the commissioner himself, because it was one of the goals to reach citizens who use mobile devices.

Public Manager

A public manager points out that mobile apps,

...seemed like the next logical step. We know that more and more people are using their phones to do conversations and socializing, and if they want information, they want it now. They are not going to wait. If they have a question at the grocery store, they are not going to wait until they go home and remember to go to the website [23] and look it up. They are going to look it up while they are standing there. So, launching the mobile version of Ask Karen seemed to be the next logical step. [23]

Public Manager

This website [23] is a service that is already hosted by a vendor, and consequently the agency outsourced the design of a mobile app to the already trusted vendor instead of acquiring internal personnel resources to rebuild the app from scratch. Similarly, patients need health-related information at their fingertips in the moment they walk into a pharmacy and need to explain their symptoms, or want to know the inspection scores of a restaurant, which are, for example, provided by the “DON’T EAT AT___” app [24]. Searches are highly situational, context specific, and results need to be reliably delivered just in time in the right format that is easy to interpret by nonprofessionals.

Overall, the first pathway mostly leads to reuse of already existing agency content to make it available on all technological platforms through which citizens access the agency’s information.

#### Pathway 2, Top-Down Policy Mandate Triggers Development

The majority of federal agencies follow the second pathway. Triggered by top-down policy mandates in the form of a presidential executive directive, a new digital strategy agenda, and follow-up guidance for the implementation of mobile apps issued by the federal CIO, agencies start to develop mobile apps. Initially, the Digital Government Strategy focused on the improvement of customer-facing services for mobile use [25]. To accomplish this goal, each federal agency had to identify two existing customer-facing services and optimize them for mobile use. It is therefore not surprising that the majority of the existing apps focus on “low-hanging fruits”, such as apps that mainly provide previously vetted information and education functionalities. An additional executive order laid out directions to make open government data available in machine-readable format so that government datasets can be accessible on data sharing websites [3,17,26]. Follow-up guidance from the CIO’s office provided agencies with best practices to create an increased awareness of the potential of mobile apps [8,27].

For most agencies, these policies triggered the first internal discussions and experiences with mobile apps, as one of the public managers explains,

It wasn’t much of a conversation to start the mobile apps. In conjunction The White House had a directive that they wanted everybody to have something mobile by the end of the year. We actually have a whole slew of tools that we will be offering over the next few years.

Public Manager

And another interview partner adds,

Now since the government has passed the Digital Government Strategy mobile will be part of your product line. Prior to that mobile was viewed as like, “Ah, it’s like a fad, or it’s something nice to have, but it’s not part of our core product line”. With our website, that we are launching, mobile will be part of that core experience.

Interview Partner

Agencies that had not previously gained experience in converting their content and services to be accessible through mobile apps needed the external intervention through a top-down mandate, and the follow-up instructions, to start the internal development process.

#### Pathway 3, Civic Hackathons and Contests

Health data contests and open innovation challenges are the third pathway government agencies are taking to build mobile apps for their agency, see for example [6,28]. The federal government is using a new policy instrument called Challenges and Prizes to promote contests on an online platform [2,29]. Contests are designed to encourage idea generation processes by involving “unlikely” audiences who are usually not in contact with the agency, who have specialized knowledge about a health area, or skills to provide solutions to public management problems.

While some agencies have very concrete needs, others use the platform to allow for a free flow of innovative ideas from citizens into the agency, as one of the public managers at NIH states,

There are two ways to (come up with innovations or solutions to problems), either unsolicited applications where we just allow the applicants to come up with the problem and the solution that they want to claim it to. And then we have other means where we have a call for applications in a given area. Say if you wanted to see more applications in say electronic health records, we have a call out saying we are interested in funding applications in these areas.

Public Manager

The outcomes of open innovation contests vary across agencies. Many agencies use contests as a way to pull citizens into their health datasets, and as a result, they create a wider awareness for the availability of the datasets. These outcomes are what Mergel et al [5] called “low hanging fruits”. The actual development of marketable health-related apps is secondary, especially because agencies are usually not allowed to promote the externally developed apps through their websites. While this is a relatively negative view on the use of contests to develop mobile health apps, the major outcomes of contests and prizes in government need to be evaluated based on their effects that are realized outside of government, and are not part of the scope of this paper. However, as an example, DHHS sees the importance in these external idea generation contests as opportunities for economic development,

There is a project called “MyCancerGenome”. A doctor was the finalist on the project. Since becoming a finalist of that project, she has actually won a number of other innovation competitions. She has been the finalist in a bunch of projects. She’s raised quite a bit of money, and as a result has been able to take a project and develop it further.

DHHS Interviewee

Another prominent example organized outside the federal government is the annual Health Data Palooza hackathon [28]. Hackathons are events—in this case—initiated by government agencies to invite large numbers of programmers to collaboratively reuse government datasets and program mobile apps. The event is designed to create health-related innovations that oftentimes result in mobile health apps. However, government organizations rarely adopt the apps created by third parties for legal reasons.

**Figure 1:**
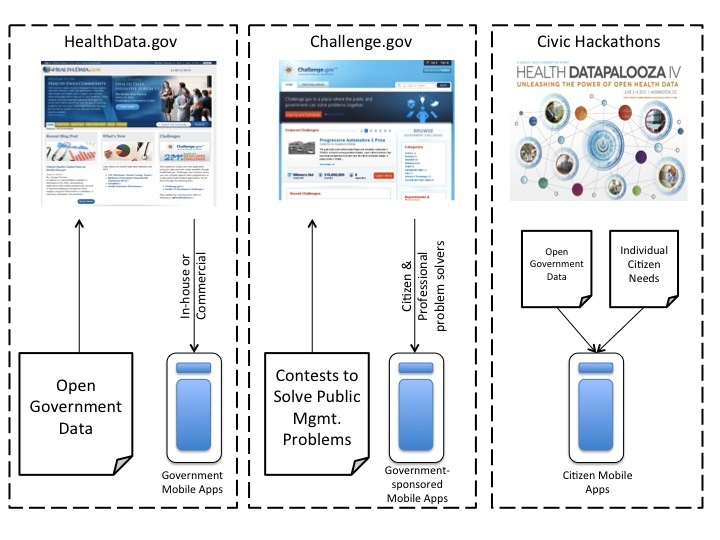
Three pathways of mobile health apps development. Mgmt. = Management.

In summary, Figure 1 shows the three main pathways of mobile health apps adoption, the data sources, and outcomes.

### Barriers for the Adoption of Mobile Apps in Government

#### Main Challenges

The main challenges of adopting and successfully introducing mobile apps in government focus on legal issues, accessibility and compliance issues, as well as the collection and protection of individual patient data. Initially, cultural and change management issues occurred in several agencies, however these were mitigated as soon as the presidential top-down mandate made discussions and decisions obsolete. The search for government information with the help of mobile apps is significantly different from the search on a desktop computer. Data need to be available in the same moment citizens need them, for example, when they are making buying decisions at a car dealership, they want to know about safety ratings; when they are about to buy new lighting or light bulbs for their house, they need the information as they are standing in the aisle of a home improvement store; or when they are listening to a warning on the radio regarding a food recall. What all of these issues have in common is that mobile apps need to deliver different types of Internet interactions with government data, searches are highly situation-based and context-specific. As a consequence, the results need to be reliable, delivered just in time in the right format, and easy to interpret.

#### Barrier 1, Legal Challenges

Legal challenges in the public health sector occur on a different scale and magnitude than in other agencies. For every step of the development—or codevelopment process with the public—public managers have to involve counselors to clarify the risks, the type of data that are published or released to the public through the app, and the way the agency is collecting data from citizens. A public manager describes the lengthy process to gain consent within his agency,

We had wanted to do [a contest] for years actually. And as a regulatory agency we live under certain laws and regulations that other agencies don’t. So, an agency like the General Service Administration GSA, National Aeronautics and Space Administration, or National Oceanic and Atmospheric Administration, who have as part of their mission, reaching out to people, to have them take advantage of the data that they have collected. Our mission is protecting human health and the environment and except under a couple of laws, we actually don’t have the legal responsibility for making that data available to the public. Now, we have always felt as an agency, we have a moral and ethical need to make that data available, and we have done so, really since the beginning of the Web. We were restricted from contests, based on our Office of General Counsel, our lawyer’s interpretation of certain regulations that we had to live under as a regulatory agency. But then Congress passed the new law that gave broad challenge authority across the entire federal government and we worked together with our office, OEI, Office of Environmental Information, with the Office of Public Affairs, with GSA, and with our general counsel, to make sure that this fit within the other regulations that we had to live with, and they approved for the first time, our doing a contest. So we launched the contest, we had a couple of codathons where we had Environmental Protection Agency (EPA) people either at the codathon, or available by phone to talk with people about the data. We deployed a number of new Web services to let people access our data securely, and then, went live and voted on the winners.

Public Manager

The outcome of using challenges and prizes to run contests then leads to the next legal challenge, when government agencies have to promote third-party mobile health apps on their official agency website. When agencies are taking the pathway to host codathons or post contests on Challenge.gov, the legal hoops they have to jump through are higher than during the development process of their own apps. While the vetting and review process is the same as for in-house apps, the agency allows the free promotion of a third-party app for a certain amount of time only. Here is how one public manager explains the process,

There were no monetary prizes in our contest, so the people who submitted with the [obligation] that, for one year after they won, the application had to be available for free. And that during that time, we would promote it on our website. But then after that time, they could surcharge with work, it was their choice after that.

Public Manager

The main legal challenge, however, occurs in the negotiations with the Apple App Store outside of government. Apple’s App Store is subject to California law, and Apple’s terms of service agreements had to be adjusted to comply with federal law. A public manager explains the process,

It took us a while to get [the app] released, but that had to do with difficulties we had with Apple’s Terms of Services. It had language in there that was not acceptable to the federal government. It took quite a while before Apple eventually added language to their standard terms of service that made it possible for us to legally get the app up in the App Store.

Public Manager

The language changes included indemnity clauses or determination which courts can handle disputes.

Agencies moving their apps to the App Store were the first to run into barriers that had to be worked out, but eventually also led to procedural changes in Apple’s app developer licensing process to accommodate the needs of the federal government. Agencies who had outsourced the app development did not experience the same challenges, because their external developers had a business license,

Our very first challenge was that when we were developing this app, we were one of the very first mobile apps to come out of the federal government, and so, even Apple did not know what to do with us when we applied for the developer license. I really wanted our app to be listed in the app store as developed by the Office of Dietary Supplements at the NIH, not by our contractor who was doing the work, because I wanted our app to have more, it just looks better. People would trust it more if the app was listed as developed by the government. Apple had never had a federal government agency apply to develop an app, they had only had a few apps from other federal government offices, but they were listed as developed by their contractor. It took about two months with Apple to get them to understand our needs were different than the needs of a private company or a private individual who was developing an app, and we, at the federal government did not have some of the documents they wanted from us, we don’t have a business license, we don’t have a contract. You know, we’re the federal government. That was one of the very first obstacles we had, until I was able to find a human being at Apple, and talk with them. They had a federal office that I didn’t even know about, and they changed a few things so that in the future people didn’t have the issues that we had.

Public Manager

#### Barrier 2, Accessibility Issues

mHealth accessibility issues emerged as one of the critical factors that had to be considered in the strategic planning, design, and development process. All federal agencies have to adhere to the accessibility guidelines for electronic and information technology as described in Section 508 of the Rehabilitation Act [30]. However, when agencies started to explore mobile health apps, there were no guidelines available specifically for mobile and smartphones, so that the existing guidelines for website development were used as guidance. The only guidance the Digital Government Strategy provides pushes agencies to develop for all mobile platforms, and test the apps before they are released to the public, “...develop secure, device-agnostic mobile applications, provide a development test environment to streamline app delivery, foster code-sharing, and validate official government applications” [25] (no page). Accessibility includes several different aspects that include a variety of citizen needs on a continuum between disability accessibility to platform preferences, “Whenever someone says accessibility in government, they generally think of Section 508, access for users of low vision, but also, considering accessibility in terms of the mobile access to government data, accessibility on different devices, and broadband access”.

Given the lack of initial guidance, public managers had to make sure that their interpretation of the laws, regulations, and current standards for Web development are applicable to mobile apps. A public manager at NIH explained,

Unfortunately, the guidelines are not really clear yet for mobile devices. So we tried to treat it like a website, and tried to make it as 508 compliant as we possibly could.

Public Manager

Compliance officers and users were invited to test the final app. A public manager described the internal process to assure that the features are accessible to screen readers,

To promote accessibility, we have been meeting with our internal 508 Accessibility Office. I’ve actually personally sat with them while they have done their testing and sat through their experience. We are big on user experience in our area, and we like to actually experience what the user experiences, so I sat with one of our employees who happens to be blind and uses a screen reader on his iPhone, and I got to experience what he experiences. We actually have things to make improvements for the next round, based on what we experienced in his feedback.

Public Manager

Another agency established Section 508 standards as the minimum,

We try to strive for better than that, because we aim for accessibility, not just mere 508 compliance. We actually have an accessibility expert on staff that has been able to train the other quality assurance testers to test not just for Section 508 compliance, but for just general accessibility. One of the biggest things you can do on any platform is use the standard user interface controls. The minute you start designing your own, creating your own button objects rather than using or even tweaking the standard button objects, you create a whole new problem for yourself. And now you have to also develop means for the screen reader to recognize and read that item. The approach I described I’d say will get you about 95 to 98% of the way there, if not all the way there. There are certain things that you can do with regards to how you order things on the screen, but we tend to solve that problem separately just by trying to use really good user experience design practices.

Public Manager

Accessibility, therefore, incorporates a broad interpretation of access by, and inclusiveness of, all citizens no matter what their technological preferences are or their capabilities to access the content.

While some mobile phone platforms already have accessibility features built in, such as magnification, other platform providers do not offer these yet, and public managers have to make decisions based on the law. If a platform does not provide the features, development is prioritized toward those platforms that comply with Section 508, giving a lower priority to platforms that lack the features,

I have been developing a lot for the Web, but with a mobile phone it was different. The phones are having those accessibility features built in, we can try to develop towards those. The problem is there are no standards yet in the accessibility space for mobile phones. My worry there is you develop for one phone, and then it doesn’t work for the way the accessibility has been done on another phone. It’s still a very evolving issue. It is one that is important. It’s important simply because we want to serve citizens on a moral and ethical level, and it’s important because we have a legal responsibility under the Section 508 of the Rehabilitation Act to design our information technology systems.

Public Manager

Given the apparent lack of standards, guidance, and the vast differences in what the individual mobile platforms are offering, it is therefore no surprise that agencies develop for the iPhone first, and later on adapt the initial app for other platforms. Another consequence is that the first round of apps is mostly text-based, does not include many interactive features, and is a safe solution to comply with the presidential mandate, as one public manager explains,

What we are trying to do is be device agnostic as much as possible. That is why for our first launch, we are making this into a mobile optimized page. All you have to do is from any mobile device, more specifically from iPhone, any Android device, or tablet, iPad and Blackberry, you open up the browser that’s in within your phone, you type in our URL, and it should automatically detect your device, and render the appropriate dimensions or size for that device.

Public Manager

#### Barrier 3, Privacy Issues

Privacy and security issues in the development process of mobile health apps play a central role for all agencies. The terms are oftentimes used in tandem without clarifying the difference. As an example, the Digital Government Strategy adopted the following language, “As good stewards of data security and privacy, the Federal Government must ensure that there are safeguards to prevent the improper collection, retention, use, or disclosure of sensitive data such as personally identifiable information (PII)” [25] (no page). Consequently, public managers tend to use the terms interchangeably. However, these are two distinct concepts. Privacy focuses on the personally identifiable information of data solicited and stored through the app from citizens, and security issues focus on the safe archiving and the prevention of improper release of the data to third parties or the public in general. Both issues are important mechanisms to manage and reduce organizational risks for the federal government according to the National Institute of Standards and Technology’s risk management framework [31].

Privacy issues arise as soon as an agency actively solicits and collects information from mobile health app users. Given that the majority of agencies are using mobile health apps to provide information to citizens simply through a different channel than the known and trusted agency website, there is usually no need to collect personally identifiable information. However, every time a citizen downloads an app from the agency’s site or through an app store, the Internet Protocol (IP) address of the phone is recognized in the same way as a visit to an agency’s website results in the delivery of the IP address to the host and a traceable record. A public manager explains the process,

We are not asking for information from [citizens], we are not asking for Global Positioning System (GPS) coordinates, so there is no reason there to have any concerns over privacy per se. Your phone is just like a desktop. I don’t think a lot a people understand that, you have an IP address. But, we are not tracking users or anything like that, we do have statistics where we have aggregate data just like we would on a website. But other than that, there’s no privacy issue.

Public Manager

A public manager at the EPA explains the process of how data from the agency that feeds into the mobile app are stored, and how the app provides it to the users. Information is only collected in direct relationship to the app itself, and the agency tracks the number of downloads, deletions from the App Store, and how often the app is used,

The way that we developed our apps is, we would load everything into a database that we have control over, which is public accessible, and then, even our apps, all accessed any information we needed with Web services that we designed, and so we designed the security into them. They would only take connections from our applications. We wanted to track usage, While I can track downloads and deletions for instance from the Apple iStore, if I wanted to track usage, I have to be able to count how many times [the app is] used. We don’t keep any information, like phone identification, or anything that would identify a person. All we keep track of is the number of times that the Web service was called.

Public Manager

Those apps that are actively helping citizens to track their own data or store individual medical information are of greater concern. The solution to the problem emerged over time, public managers decided to develop apps so that personally identifiable information is only stored on the citizen’s phone without transmission to the agency. Responsibility to protect citizens’ privacy and security of their own data is therefore put on the shoulders of the users themselves. A public manager explained that they looked for evidence in other corners of the government system, and replicated those efforts,

There is a bit of ambiguity right now, whether, having the person’s full name and email address, even if voluntary provided, is considered a personally identifiable collection of information, a system of records. It makes it pretty challenging, but we figured that there were precedents in terms of public comment on regulations online, and folks participated in the White House’s social media sites, and they also provide email addresses and names.

Public Manager

However, as mobile experiences are evolving, and agencies start to experiment with higher levels of interactions, more complex data combinations, and more features for their apps, more privacy issues will have to be resolved,

When we came out with what we call our version 1 of our app that version only lived locally on someone’s device. So that there were no security issues, because any information that they put into the app would only stay on their phone. We never saw it, we couldn’t see it, it didn’t sync, anything like that. We have since as of this past winter launched version 2 of our app, and this new version rather than being a native app in the Apple Store, it is a mobile web app, and it can sync between devices from Apple to Android to Blackberry to iPad, to desktop, to iPad, all over the place. In order to be able to sync like that between devices, and to have people’s data synced, the user has to create an account, with their username, which is their email address, and a password. That means that we are now storing their data in the cloud so to speak. All of a sudden we had privacy issues that we had to think about. We weren’t collecting personal identifiable information, but everything was being encrypted, so we can’t see what a user, what dietary supplements the user is taking for example.

Public Manager

Working in collaboration with the agency’s privacy officer, the solution was to encrypt citizen data.

Overall, agencies that only use mobile health apps to recycle content from their website, and do not track or pull in information from citizens, are not concerned with privacy issues. This topic only becomes relevant when agencies open their own datasets and let citizens reuse data. In summary, data collection from citizens falls into six different categories and depends on the type of app, all apps collected IP addresses. Apps providing answers based on locations ask for the submission of longitude and latitude data to match the GPS location with government scores, for example, for sanitation scores. Apps that are designed to alert citizens of impeding risks, for example, for dietary supplements or food recalls, are oftentimes designed so that citizens have to opt in and names, email addresses, as well as the location of the phone are submitted to the agency. Apps with higher levels of interactions, for example, apps that are tracking patient data are usually storing the citizen input directly on the phone, and therefore prevent all privacy issues that might arise in case data are transmitted to the agency. The following table provides an overview of the type of data entry, the data collected by each app, and where the data are stored.

**Table 1:** Summary of personally identifiable information collected through mobile health apps.

Type of data entry	Collected by type of app	Data stored
IP address	Collected by all apps	Submitted to agency
Longitude, latitude (GPS location)	Ask for submissions (for example, feedback on sanitation scores, check ins)	Submitted to agency
Email address, name	Apps issuing alerts	Submitted to agency
Patient data (symptoms, medication, dietary supplements)	Apps tracking intake, dosage, phases of symptoms, changes over time	Stored on phone only
Individual health information (smoking habits, eating/intakes, physical activity)	Apps promoting behavioral changes	Stored on phone only
Individual scores	Game apps	Stored on phone, opt-in to share with social networks

#### Barrier 4, Security Issues

Security concerns include two dimensions, first, government provided content needs to be stored securely, and if possible, in a separate database that can be continuously accessed by mobile apps used by the public. Second, for those apps through which citizens are submitting information to the government, these data points need to be encrypted and safeguarded. The Digital Government Strategy provides guidance for authorization and encryption of agency data provided to the public, but leaves the protection of citizen data and devices as a responsibility of the citizens, “data owners can focus more effort on ensuring the safe and secure delivery of data to the end customer and fewer resources on securing the device that will receive the data” [25] (no page).

The main goal is to reduce the risk for government itself, and agencies are following OMB's risk assessment instructions to protect personally identifiable information [32]. Web sites and mobile apps are going through a rigorous security process, as well as periodic reviews. However, as outlined earlier in the paper, agencies only adopt one-way interactions (citizens with government data), mostly decided not to ask for user data, avoid e-transactions, and focus on the protection of their own content in-house, as the NIH interview partner highlights,

It’s all the information is on a server here at the NIH. So it’s very protected. It’s not just out there.

Interview Partner

The biggest obstacle occurs for those agencies that are providing apps that collect data from citizens on a voluntary basis; however, the solution is similar to the previously discussed privacy issues,

The users’ data that they’re recording needed to be stored. One option was to create a Web service on our end, on our servers that would store this data. However that raised a lot of security concerns, in the sense that now we have to actually build in a lot a extra security for this to protect that data on our infrastructure. What we decided to do was to have the user store that data locally. In addition to resolving the security and privacy issues, it also made things a lot snappier for the user, because now they didn’t have to reach out to a server, or necessarily have Internet access in order to use the app to just store data locally.

Public Manager

Agencies that are planning for more complex apps in the future need to include solutions to meet these needs,

We do know that there’s going to be a need at some point for the apps to reach out and grab or store personal data, so that’s why on our end we got an API that’s on our developer site. One of the current projects we have got right now is to add authentication capabilities using Open ID, etcetera. So that if we need to either make data available that requires authentication, or we have data sets that are read right, such as one that where an app can actually say, send data to us, we can do so securely.

Public Manager

Others stay on the lowest level of adoption and rely on the existing security protection, and do not veer away to iPhone or Android platforms, instead, relying on Blackberry that already provided the needed security checks,

Security just depends on the application itself. We haven’t moved away from Blackberry, and that’s been our standard for some time. Until we do, I don’t know of any major security issues any more than if you do a web application.

Public Manager

## Discussion

### Solving the Paradox

Solving the paradox of openness, customer-centric design of health-related mobile apps, and the federal policy to prevent the collection, retention, use, or disclosure of sensitive data, such as personally identifiable information, has resulted in 33 DHHS-sponsored mHealth apps [25] (no page). Most of the apps, however, are so-called native apps that are replicating the Web presence of content public agencies already provide to their stakeholders through their regular e-Government presence. These findings are in line with Cucciniello and Nasi’s findings [33] that show that public health innovations are especially challenging governments, and that medical apps lack quality.

This push tactic of disseminating information to the public is fully in line with the agency’s mission, as the following statement of an interview partner from the Office of Dietary Supplements at the NIH says,

Our office supports research on dietary supplements, and then we disseminate the results of that research out to the public. We try to get more science-based information about dietary supplements out to the American public.

Interview Partner

Besides other established dissemination channels, such as newsletters or the website itself, apps allow the agency to actively push the information to the citizens, instead of waiting for citizens to stumble upon the information during a Web search.

This study shows for the first time—to the best of the author’s knowledge—the strategic and managerial decisions that have to be made before government agencies are able to even experiment with these simple apps that purely focus on representation, education, and informing the public. Previous research on the adoption of new technologies in government has shown that adoption patterns usually follow a similar adoption curve, first, government agencies start with simple versions of the new platforms, and, in the process, work out all the internal barriers, such as uncertainty about legal issues and considerations on how to apply the existing regulations, such as the compliance with Section 508 of the Rehabilitation Act or data protection and security issues, see for example [19]. What is surprising is that none of the interview partners pointed to restrictions of the Health Insurance Portability and Accountability Act. Instead, they simply avoided the collection of patient data, and handled mobile app development in a conservative manner.

Only after the internal issues have been satisfactorily determined, and existing rules are adapted to the new technology standards, agencies might be willing to explore more complex versions of new technologies, in this case interactive mobile health apps [10,11,19]. What is currently observable in this specific sample is the experimentation of what Rogers labeled “early adopters”, who served as internal trailblazers and saw initial opportunities to start transforming their agencies content to a new technological platform [34]. With the introduction of an external intervention in the form of a new top-down policy, the majority of agencies followed the mandate, but not necessarily out of an internal need, other successful apps, or a push from citizens to move into mobile.

This article, therefore, contributes to the existing literature on new technology adoption in government and provides microlevel insights into the internal decision-making processes that lead to the adoption of mobile apps, and, by that, helps to open the black box of government.

### Limitations

This study focused on a very specific sample, federal-level agencies in the US Government in their early adoption phases of mobile health apps. It excluded by design other levels of government, as well as citizen apps, that are not hosted or promoted by the government. Citizen-designed apps are not subject to the same regulations and standards as federal government agencies. Innovations, especially when it comes to data entry, tracking, and sharing, are therefore much more prevalent in other sectors, but were not part of this study, and the insights are therefore limited to this specific sample.

## Abbreviations

CDC: Centers for Disease Control and Prevention
CIO: Chief Information Officer
DHHS: Department of Health and Human Services
EPA: Environmental Protection Agency
GPS: Global Positioning System
GSA: General Service Administration
IP: Internet Protocol
NIH: National Institutes of Health
